# Atrial natriuretic peptide may inhibit motion sickness through reducing aldosterone-induced increase in endolymph volume of the inner ear

**DOI:** 10.1371/journal.pone.0359426

**Published:** 2026-09-25

**Authors:** Qian-Cheng Lu, Wei Ji, Xia Li, Jian-Gang Ge, Li-Hua Xu, Zheng-Lin Jiang

**Affiliations:** 1 Institute of Special Environmental Medicine, and Co-innovation Center of Neuroregeneration, Nantong University, Nantong, Jiangsu, China; 2 Department of Radiology, The Second People’s Hospital of Nantong and Affiliated Rehabilitation Hospital of Nantong University, Nantong, Jiangsu, China; Southern University and A&M College, UNITED STATES OF AMERICA

## Abstract

Previous studies have suggested that aldosterone is potentially involved in the development of motion sickness and that atrial natriuretic peptide (ANP) may inhibit it. Therefore, the present study was conducted to investigate the inhibitory effects of ANP on aldosterone activity in motion sickness and on aldosterone-induced changes in endolymph volume in the inner ear. We found that rotatory stimulation induced an elevation in plasma aldosterone levels, intraperitoneal aldosterone injection caused motion sickness-like responses in both guinea pigs and mice, and ANP inhibited these responses. Moreover, aldosterone injection induced an increase in inner ear endolymph volume in guinea pigs, whereas ANP alleviated this endolymph expansion. In addition, mineralocorticoid receptor (MR) expression was increased in the inner ear tissues after rotatory stimulation and aldosterone injection, and in cultured vestibular epithelial cells after aldosterone treatment, whereas ANP reduced MR expression. In cultured vestibular epithelial cells, ANP reduced the aldosterone-induced nuclear distribution of MR, as well as the expression of ENaC and Na^+^-K^+^-ATPase and the membrane distribution of Na^+^-K^+^-ATPase. Furthermore, ANP inhibited the aldosterone-induced increase in the intracellular K^+^ concentration in cells incubated with a simulated endolymph fluid, and the increase in intracellular Na^+^ concentration in cells incubated with an extracellular solution. In conclusion, the present results suggest that ANP may play an anti-motion sickness role by reducing the plasma aldosterone levels and inhibiting aldosterone-induced endolymph expansion by downregulating downstream target protein expression and activities, thereby altering the resultant ion concentrations in the inner ear epithelial cells.

## Introduction

Motion sickness poses significant challenges to human spaceflight, aviation, and nautical operations, as well as to travel, and visual tasks performed under virtual reality conditions; however, the exact underlying mechanisms and biological significance remain unclear [1,2]. Currently available drugs for motion sickness are only partially effective and are associated with unwanted side effects, such as dry mouth/eyes, blurred vision, photosensitivity, dizziness, headache, drowsiness, and sedation; in particular, the inhibitory effects on the central nervous system (CNS) exerted by commonly used anticholinergic and antihistamine drugs pose considerable concerns for people working at sea or in space [1–4]. Therefore, further research is warranted to elucidate the pathophysiological mechanisms of motion sickness, identify novel therapeutic targets, and develop new anti-motion sickness agents devoid of CNS side effects.

Accumulating evidence suggests that circulating levels of the mineralocorticoid aldosterone are elevated in association with the occurrence of motion sickness. As early as 1985, Stalla et al. reported that serum aldosterone levels in participants increased progressively with the duration of Coriolis stimulation administered via a rotary chair [5]. Dai et al. similarly reported that circulating aldosterone levels in pilots were significantly elevated under Coriolis stimulation [6]. Grigoriev et al. reported that plasma levels of aldosterone, renin and other hormones in human subjects were significantly elevated following vestibular stimulation [7]. Pei et al. reported that urinary levels of hormones such as aldosterone were significantly elevated under optokinetic stimulation [8]. In addition, other studies have demonstrated an increase in renin-angiotensin-aldosterone system activity and a significant elevation in circulating aldosterone levels during spaceflight [9–11]. Collectively, these findings suggest that aldosterone may play a role in the development of motion sickness.

As a mineralocorticoid, aldosterone is not only involved in the systemic water and electrolyte balance and blood pressure maintenance, but also in the regulation of endolymph volume and ionic balance in the inner ear by modulating the activities of its corresponding target proteins. The inner ear is generally regarded as the anatomical basis underlying the pathogenesis of motion sickness. Numerous studies have demonstrated the expression and distribution of mineralocorticoid receptors (MRs) in the inner ear [12–14], as well as the capacity of aldosterone to bind these MRs [15]. In addition, Na^+^-K^+^-ATPase (NKA) and the epithelial sodium channel (ENaC) are well-established downstream target proteins of aldosterone. NKA is distributed throughout multiple compartments of the inner ear in both humans and rodents [16–18], and ENaC is likewise expressed in the inner ear [19–22]. The activities of both NKA and ENaC are modulated by aldosterone [23–26]. Therefore, aldosterone-mediated regulation of inner ear water and electrolyte balance under conditions of elevated circulating aldosterone may contribute to the induction of motion sickness.

Numerous studies have demonstrated that intraperitoneal injection of aldosterone induces membranous labyrinth hydrops in the inner ear of guinea pigs [27–29], confirming that aldosterone exerts a direct effect on the increased endolymph volume. Recently, we reported that an increase in endolymph volume in the inner ear after rotatory stimulation was potentially involved in the development of motion sickness, and that attenuation of this endolymph volume increase by atrial natriuretic peptide (ANP) was able to inhibit motion sickness [30]. Therefore, we hypothesize that vestibular stimulation-induced elevation of circulating aldosterone levels may disrupt inner ear endolymph homeostasis and thereby contribute to the development of motion sickness. However, it remains unclear whether ANP could interfere with the mineralocorticoid signaling pathway, inhibit the effects of aldosterone on the endolymph volume, and consequently, attenuate motion sickness. The present study was thus designed to investigate the inhibitory effects of ANP on aldosterone activity in motion sickness, as well as the associated changes in endolymph volume and the activity of the mineralocorticoid signaling pathway in the inner ear.

## Materials and methods

### Animals and chemicals

Guinea pigs (body weight 300–350 g), ICR mice (body weight 20–22 g), and ICR mouse pups at postnatal day 8 (P8) were obtained from the Experimental Animal Center of Nantong University, Nantong, China. Adult animals were housed under a 12-h light/12-h dark cycle (light, 08:00–20:00h; darkness, 20:00–08:00h) at room temperature (22–24^°^C) with free access to standard rodent chow and water. Animal health and behavior were monitored daily. All procedures in this study were conducted in accordance with the institutional guidelines, which comply with the ARRIVE (Animal Research: Reporting of *In Vivo* Experiments) guidelines and were approved by the Institutional Animal Care and Use Committee, Nantong University (approval number S20220222-029 for guinea pigs and S20220222-027 for mice). Blood sampling and inner ear tissue dissection of animals were performed under anesthetized condition, after which the animals were immediately euthanized by overdose of anesthetic, with no animals dying before meeting the criteria for euthanasia. Each experimenter had completed a training course in experimental animal science including animal care and handling.

Common inorganic salts were purchased in China. Culture medium (DMEM, Lot No. MA0212) was purchased from Meilunbio (Dalian, China). ANP (peptide 1–28, Lot No. A407225) was purchased from Alatin (Shanghai, China), and aldosterone (Lot No. 15273) from HWRK Chemical (Ann Arbor, USA). The following primary antibodies were purchased: monoclonal anti-β-actin (Lot No. 66009–1-lg-100ul) and anti-NKAα1 (Lot No. 14418–1-AP) from Proteintech (Chicago, USA), anti-MR (Lot No. rMR1–18 1D5) from Developmental Studies Hybridoma Bank (Lowa, USA), anti-zonula occludens-1 (ZO-1, Lot No. sc-33725) from Santa Cruz Biotechnology (Dallas, USA), and anti-ENaCα (Lot No. ASC-030) from Alomone (Jerusalem, Israel). CoroNa^TM^ green-AM (Lot No. C36676) and ION potassium green 2-AM (Lot No. ab142806), two fluorescence indicators for Na^+^ and K^+^, were purchased from Invitrogen (Carlsbad, USA) and Abcam (Cambridge, UK), respectively. Poly-D-lysine, cytosine β-D-arabinofuranoside, sodium dodecylsulfate and other chemicals not specified elsewhere were purchased from Sigma-Aldrich (St. Louis, USA).

### Rotatory stimulus

A rotatory stimulator designed for animal use was constructed according to the protocol described by Crampton and Lucot [31]. All animals were rotated in the stimulator using a paradigm of alternating accelerations and decelerations. The acceleration rate was 16^°^/s^2^ for 7.5 s with a maximal velocity of 120^°^/s, after which the corresponding deceleration rate was 48^°^/s^2^ for 2.5 s. The clockwise and counterclockwise rotations were alternately repeated for 120 min, as described in our previous study [32].

### Conditioned taste aversion induction via intraperitoneal injection of aldosterone

Conditioned taste aversion (CTA) was used as an alternative indicator of motion sickness-like response induced by aldosterone injection [30]. To induce CTA, guinea pigs or mice were intraperitoneally injected with aldosterone at a dose of 100 μg/kg or 114 μg/kg, respectively. The aldosterone doses were based on published literature [33,34] and our preliminary data (S1 Fig). Prior to aldosterone injection, the animals were provided with both tap water, and 0.15% sodium saccharin solution (SSS; Northern Food Co., Ltd, Tianjin, China) as a novel fluid for 48 h. Following aldosterone injection, SSS access was maintained for 24 h. The SSS intake volume per 24 h was recorded. A reduction in SSS intake volume after aldosterone injection was regarded as successful induction. ANP was intraperitoneally injected at a dose of 400 μg/kg for guinea pigs and 500 μg/kg for mice, 30 min prior to aldosterone injection; the inhibitory effect of ANP on CTA induction was then assessed. The ANP doses were based on our previous study [30] and published literature [35,36].

### Swimming test for evaluating vestibular function

The swimming test was performed at a fixed time of day. Vestibular function in guinea pigs was assessed by measuring the curvature of swim trajectory in a Morris water maze [30]. The apparatus consists of a circular black pool 60 cm in height and 180 cm in diameter, a hidden platform, and a video/computer tracking system. The maze was filled with water maintained at 21–25^°^C to a depth of 40 cm prior to each trial. Guinea pigs were habituated to the behavioral testing room for at least 30 min prior to testing. Training trials were performed once per day for seven consecutive days. For each training trial, the hidden platform was removed; each animal was placed into the pool at the center facing the wall; and 10 head-tracking traces per animal were recorded using ANY maze software (Stoeling Co., Wood Dale, USA). After each trial, the animals were towel-dried. Animals that failed to swim in a straight line on the seventh day were excluded from subsequent experiments. The treatment trial was performed on the eighth day; the experimenter was blinded to the treatment groups; and the average value of five trials per animal was used for analysis. The open-source soft Fiji plugin “Kappa” was used to quantify the curvature of the swim trajectories which was also used as an alternative indicator of motion sickness-like response. ANP was intraperitoneally injected at a dose of 400 μg/kg, 30 min prior to aldosterone injection; the effects of ANP on the curvature of the swim trajectory at 3 h after aldosterone injection was then assessed.

### Enzyme-linked immunosorbent assay (ELISA)

Blood was collected from the right ventricle of guinea pigs within 1 h after cessation of rotatory stimulation, under isoflurane inhalation anesthesia. Blood samples (2 mL of each) were placed in test tubes containing 200 μg of dried sodium heparin, gently mixed, and immediately centrifuged at 2,000 rpm at 4˚C for 5 min. Plasma was then collected and stored at −80˚C until further analysis. Plasma aldosterone levels were measured using an aldosterone ELISA kit (Lot No. E-EL-0070c, Elabscience Biotechnology Co., Wuhan, China) according to the manufacturer’s instructions. ANP was intraperitoneally injected at a dose of 400 μg/kg in guinea pigs, 30 min prior to the rotatory stimulation; the effect of ANP on plasma aldosterone levels after rotatory stimulation was then assessed.

### Measurement of endolymph volume

Magnetic resonance imaging (MRI) of the longitudinal sagittal plane of the guinea pig’s head was performed under general anesthesia with 2.5% Avertin (15 mL/kg, i.p.) [30]. Gadopentetate dimeglumine solution (280 mg/kg, Lot No. 20210512, Consum Pharmacy, Guangzhou, China) was intravenously administered 90 min before induction of anesthesia and 4 h before the MRI scan. All guinea pigs were imaged using an MRI scanner (Ingenia 3.0 T, Philips, Amsterdam, Netherlands) with a 4-channel animal head coil. The T2WI-DRIVE-HR sequence was selected (repetition time/echo time, 1550 ms/251.8 ms; matrix, 124 × 149; flip angle, 90°; slice thickness, 0.2 mm; total slices, 200; average scanning time, 21 min 31 s; field of view, 50 mm × 50 mm) to delineate the anatomy of the entire fluid-filled compartments. Perilymph was highlighted by the gadolinium contrast agent, while the non-enhanced (shaded) region represented endolymph, as gadolinium contrast agent has limited access to the endolymphatic space. Data were processed offline using ImageJ Fiji software. A region of interest encompassing the inner ear was defined using a square bounding box on the full head MRI images. Using ImageJ Fiji software, the areas of the non-enhanced (endolymph) region and the total fluid-filled region in the cochlea were measured according to published literature [37]; the ratio of endolymph volume (number of layers × layer thickness × non-enhanced area) to the total fluid volume (number of layers × layer thickness × total fluid-filled area) in the cochlea was then calculated. The analyst was blinded to the treatment groups; the measurements were subsequently independently verified by a skilled MRI expert who cross-referenced the treatment groups with the corresponding images. MRI scans were initiated 3 h after aldosterone injection (100 μg/kg, i.p., S1 Fig) and ANP was administered (400 μg/kg, i.p.) 30 min prior to aldosterone injection.

### Immunofluorescence staining of frozen inner ear tissue sections

Mice were perfused transcardially with 0.9% saline followed by 4% paraformaldehyde (PFA) in 0.1 M phosphate-buffered saline (PBS, pH 7.4) under 2.5% Avertin anesthesia (15 mL/kg, i.p.). The inner ear tissues were dissected, post-fixed in 4% PFA overnight at 4˚C, cryoprotected sequentially in 20% and 30% sucrose/PBS, embedded in OCT compound (Lot No. 4583, SAKURA, USA), and sectioned at 10 μm using a cryostat. For immunofluorescence, the frozen sections were permeabilized with 0.3% Triton X-100 for 30 min, blocked with 5% donkey serum for 1 h, and incubated overnight at 4˚C with a primary antibody against mouse MR (1:100 dilution). A negative control in which the primary antibody was omitted was included in each experiment. Sections were subsequently incubated with Alexa Fluor 488-conjugated donkey anti-mouse secondary antibody (1:1000 dilution; Jackson ImmunoResearch, West Grove, USA) for 2 h at room temperature. Immunostained sections were imaged at room temperature using a laser scanning confocal microscope (TCS SP8; Leica Microsystems, Wetzlar, Germany), and images were processed using LAS X 2.0 software (Leica Microsystems).

### Primary culture of vestibular epithelial cells

Primary culture of inner ear vestibule epithelial cells was performed based on previous published protocols [30,38]. Mouse pups at P8 were used for primary culture; following euthanasia by CO_2_ asphyxiation in a transparent acrylic chamber, their temporal bones were removed and placed in ice-cold Hanks’ balanced salt solution (Lot No. C3571-0500, VivaCell, Shanghai, China). The inner ear vestibules, including the utricle and saccule, were carefully isolated under a dissecting microscope, divided into small pieces, and digested in PBS containing 0.125 mg/mL collagenase IV and 0.125% trypsin-EDTA at 37^°^C for 8 min. Digestion was terminated by the addition of three volumes of complete culture medium. The tissue fragments were then mechanically dissociated by repeated pipetting, followed by centrifugation. Following supernatant removal, the pellet was resuspended in complete culture medium and plated onto poly-D-lysine-coated coverslips for explant cultures. The explants were cultured at 37^°^C in a humidified 5% CO_2_ incubator. The culture medium consisted of DMEM supplemented with 15% fetal bovine serum, 85 μg/mL penicillin-streptomycin, and 100 ng/mL epidermal growth factor; the medium was replaced after the first 24 h, followed by half-volume medium changes every 2 days. After 7 days *in vitro*, the cultured vestibular epithelial cells were used for subsequent experiments (S2 Fig).

### Western blot analysis

Inner ears were dissected from the temporal bones of mice anesthetized with Avertin and lysed in tissue lysis buffer (Lot No. P0013B, Meilunbio, Dalian, China) supplemented with a protease inhibitor cocktail. The lysates were homogenized and centrifuged at 12,000 rpm for 15 min at 4˚C. Protein concentrations of the supernatants were determined by the bicinchoninic acid assay. Equal amounts of protein (40 μg per lane) were loaded on a 10% sodium dodecyl sulfate-polyacrylamide gel, electrophoresed, and transferred onto polyvinylidene difluoride membranes (Merck Millipore, Temecula, USA). The membranes were blocked with 5% non-fat milk in Tris-buffered saline containing 0.1% Tween-20 for 2 h at room temperature, followed by overnight incubation at 4˚C with primary antibodies against mouse MR (1:100 dilution) and mouse β-actin (1:5,000 dilution). Subsequently, the membranes were incubated with a horseradish peroxidase (HRP)-conjugated secondary antibody (1:6,000 dilution; Biosharp, Beijing, China) for 2 h at room temperature; the immunoreactive bands were then visualized using enhanced chemiluminescence reagents (Vazyme, Nanjing, China) and imaged using a Tanon 5200 imaging system (Tanon, Shanghai, China). Protein expression levels were quantified using ImageJ Fiji software and normalized to β-actin levels in the same lane (S3 Fig). All experiments were conducted in at least two independent replicates.

### Quantitative real-time polymerase chain reaction (qRT-PCR)

Inner ears were dissected from the temporal bones of mice under Avertin anesthesia. The inner ears and cultured vestibular epithelial cells were homogenized in TRIzol reagent (Lot No. 15596018, Thermo Fisher Scientific, Carlsbad, USA), and total RNA was purified. RNA was reverse-transcribed into cDNA using the HiScript III reverse transcriptase kit (Lot No. R323-01, Vazyme Biotech, Nanjing, China) following the manufacturer’s instructions. qRT-PCR was performed on a StepOnePlus PCR system (Thermo Fisher Scientific) under the following conditions: an initial denaturation step at 95˚C for 5 s, followed by 40 cycles of denaturation at 95˚C for 15 s, annealing at 60˚C for 30 s, and extension at 72˚C for 20 s. The cDNA quantity per sample was measured using a SYBR Green master mix (Lot No. Q141-02, Vazyme Biotech, Nanjing, China) for gene expression analysis. All data were normalized to the expression of the housekeeping gene *Actb* using the 2^–ΔΔCt^ method. The primer sequences were as follows: *Nr3c2*, forward, 5’-CAACTATCTGTGTGCTGGAAGA-3’; and reverse, 5’-CCTTGGTAGGAGCAATGTATGT-3’; *Actb*, forward, 5’-ACACCCGCCACCAGTTC-3’; and reverse, 5’-TACAGCCCGGGGAGCAT-3’.

### Cellular immunofluorescence imaging

The cultured vestibular epithelial cells were fixed with 4% PFA at room temperature for 15 min, followed by permeabilization with precooled methanol at −20˚C for 8 min. After blocking with 5% donkey serum for 1 h, the cells were incubated overnight at 4˚C with primary antibodies against mouse MR (1:100 dilution), rabbit ENaCα (1:500 dilution), rabbit ZO-1 (1:200 dilution) and mouse NKAα1 (1:500 dilution). For each primary antibody, a negative control was included in which that antibody was omitted. Subsequently, the cells were incubated with Alexa Fluro 488-conjugated donkey anti-rabbit or anti-mouse secondary antibodies (1:1000 dilution, Jackson ImmunoResearch, West Grove, USA) for 1–2 h at room temperature. Immunostained cells were imaged at room temperature using a laser scanning confocal microscope (TCS SP8, Leica Microsystems) and processed using LAS X 2.0 software (Leica Microsystems).

### Measurement of K^+^ and Na^+^ concentrations in vestibular epithelial cells

The cultured vestibular epithelial cells were washed three times with either a simulated endolymph fluid (containing 150 mM KCl, 5 mM NaCl, 2 mM KH_2_PO_4_, 1 mM MgCl_2_, 3 mM glucose, 25 mM HEPES, 1 g/L albumin) for K^+^ measurement or an extracellular fluid (Lot No. C0216, Beyotime, Nantong, China) for Na^+^ measurement, respectively. Either the simulated endolymph fluid containing IONPotassium Green 2-AM (5 μM) or the extracellular fluid containing CoroNa^TM^ Green-AM (10 μM) was added to the culture dish at a volume of 200 µL, and the cells were incubated with K^+^ or Na^+^ fluorescent indicator for 30 min or 1 h, respectively, at 37^°^C in a humidified 5% CO_2_ incubator. Following this incubation, the cells were washed three more times with the simulated endolymph fluid or the extracellular fluid, and live-cell imaging was performed using a live-cell stage insert on a laser scanning confocal microscope (TCS SP8, Leica Microsystems). The peak excitation and emission wavelengths of IONPotassium Green 2-AM were 526 nm and 546 nm, respectively. The peak excitation and emission wavelengths of CoroNa^TM^ Green-AM were 492 nm and 516 nm, respectively. One image was acquired every 30 s before and after each treatment, including high-concentration KCl (3 M) or NaCl (290 mM), aldosterone (1 nM), ANP (100 nM), and ANP (100 nM) plus aldosterone (1 nM). ANP and aldosterone were dissolved in the simulated endolymph fluid or the extracellular fluid, respectively. Fluorescence intensity was analyzed offline using ImageJ Fiji software. The average fluorescence intensity of 8–10 cells was calculated in each of the 3 high-magnification fields of view per culture dish, and the experiment was independently repeated once. Changes in ion concentration were reflected by the average fluorescence intensity and expressed relative to the baseline values before each treatment.

### Statistical analysis

All data are presented as the mean ± s.e.m. One-way ANOVA was used for the comparison of data from three or more group design experiments, two-way ANOVA for the comparison of data of ion concentration measurements, and the least significant difference test was used for post hoc comparisons. Differences were considered statistically significant at a level of *P* < 0.05.

## Results

### ANP inhibits both rotatory stimulation-induced elevation of plasma aldosterone levels and aldosterone-induced increase in endolymph volume and signs of motion sickness

**Fig 1:**
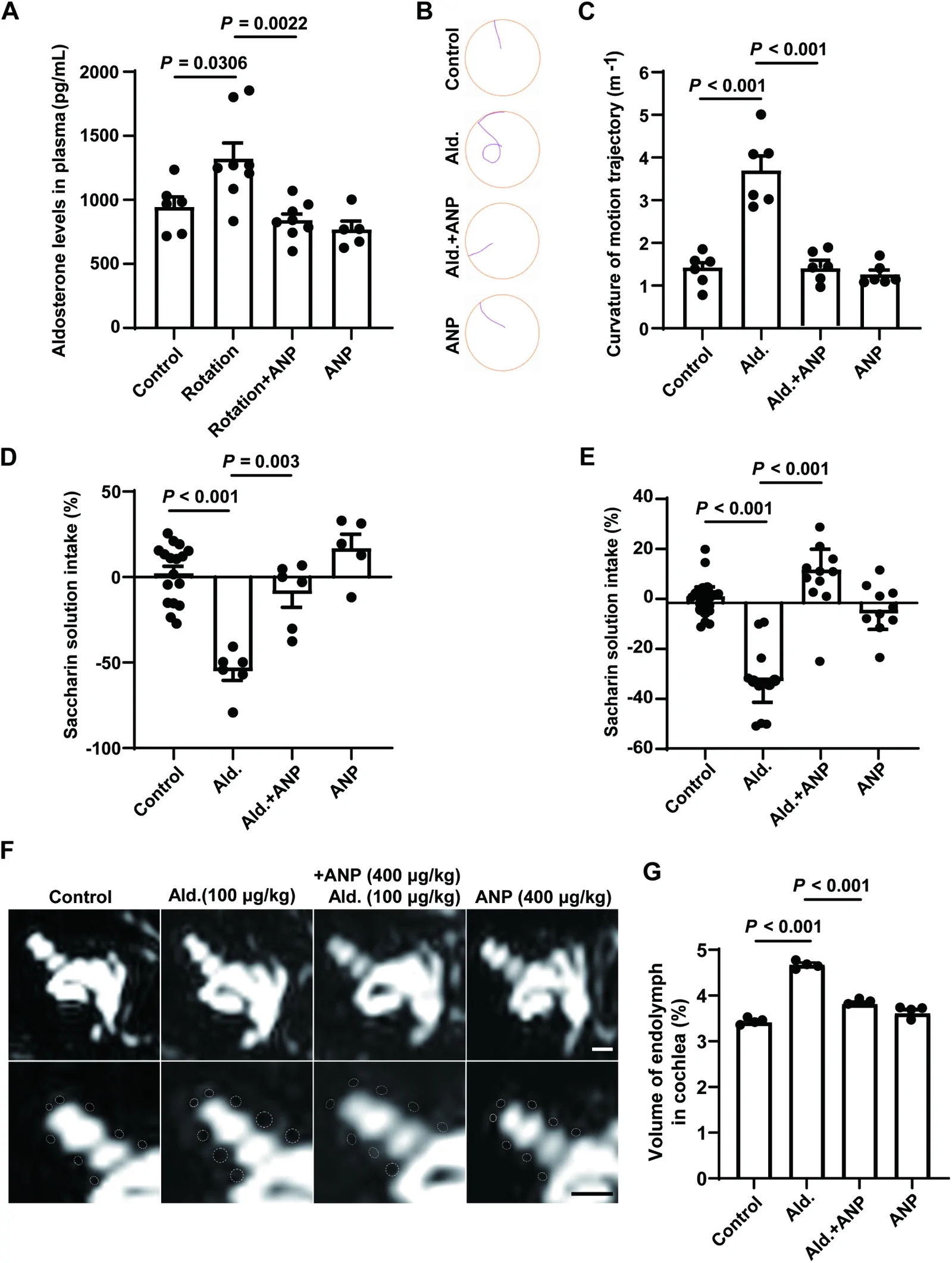
Rotatory stimulation-induced elevation in plasma aldosterone levels and aldosterone-induced increase in endolymph volume and motion sickness-like responses. A, Plasma aldosterone levels in guinea pigs (n = 5-8 animals per group). B, Representative swim trajectories of guinea pigs. C, Curvatures of the swim trajectories of guinea pigs (n = 6 animals per group). D, Changes in 0.15% SSS intake in guinea pigs (n = 5-17 animals per group). E, Changes in 0.15% SSS intake in mice (n = 10-19 animals per group). F, Representative cochlear MRI images from each experimental group of guinea pigs. The bottom row shows magnified views of the cochlear region from the upper row images; dashed circles delineate the endolymph areas (the dark parts). Scale bar, 1 mm. G, Quantification of the endolymph volume relative to the total cochlea lymphatic fluid volume (n = 3-4 animals per group). Ald., aldosterone.

As shown in Fig 1, the motion sickness-provoking rotatory stimulation significantly induced an elevation in plasma aldosterone levels in guinea pigs (*P* < 0.05, Fig 1A), whereas ANP pretreatment inhibited this elevation in plasma aldosterone levels (*P* < 0.01, Fig 1A). To further elucidate the role of aldosterone in the development of motion sickness, we investigated whether exogenous aldosterone administration could induce CTA behavior in both guinea pigs and mice, and whether it could exert any effect on the endolymph volume in the inner ear of guinea pigs. Consequently, we found that intraperitoneal injection of aldosterone induced an increase in the curvature of the swim trajectory (*P* < 0.01, Fig 1B-C) in guinea pigs and a reduction of 0.15% SSS intake in both guinea pigs (*P* < 0.01, Fig 1D) and mice (*P* < 0.01, Fig 1E); however, ANP pretreatment markedly inhibited these aldosterone injection-induced changes (*P* < 0.01, Fig 1B-E). In addition, similar to rotatory stimulation, aldosterone injection likewise induced an increase in the inner ear endolymph volume (*P* < 0.01, Fig 1F-G); however, ANP pretreatment significantly attenuated this aldosterone injection-induced endolymph volume increase (*P* < 0.01, Fig 1F-G), a finding consistent with our previous report in which ANP pretreatment attenuated rotatory stimulation-induced increase in the inner ear endolymph volume and behavioral changes in CTA and vestibular function [30].

### ANP inhibits both rotatory stimulation- and aldosterone administration-induced MR expression in both inner ear tissues and cultured vestibular epithelial cells

**Fig 2:**
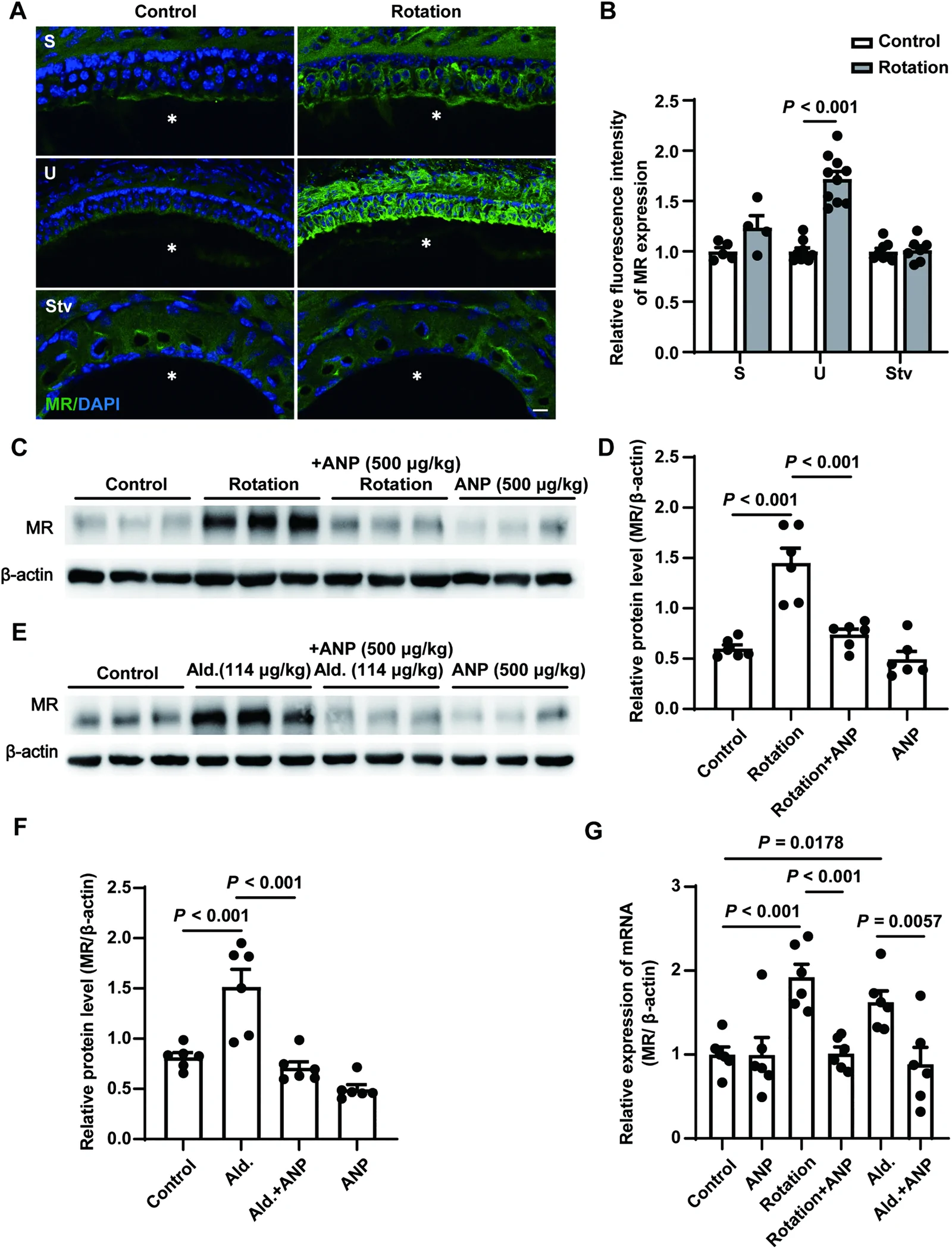
Rotatory stimulation- and aldosterone administration-induced upregulation of MR expression in the mouse inner ear tissues. A, Representative immunofluorescence images of MR expression in the saccule (S), utricle (U), and stria vascularis (Stv). An asterisk indicates the endolymphatic space. Scale bar, 10 µm. B, Quantification of MR immunofluorescence intensity in the inner ear tissues of mice subjected to rotatory stimulation or sham treatment (n = 4-10 slices from 2-3 animals per group). C, Representative Western blot analysis of MR expression in the inner ear tissues of mice after rotatory stimulus, with or without ANP pretreatment. D, Quantification of MR expression (n = 6 lanes from 3 animals per group). E, Representative Western blot analysis of MR expression in the inner ear tissues of mice after aldosterone treatment, with or without ANP pretreatment. F, Quantification of MR expression (n = 6 lanes from 3 animals per group). G, MR mRNA level in the inner ear tissues of mice subjected to rotatory stimulation or aldosterone treatment, with or without ANP pretreatment (n = 6 animals per group). Ald., aldosterone.

To elucidate the mechanisms underlying the aldosterone-induced increase in the inner ear endolymph volume, its relationship to motion sickness, and the inhibitory action of ANP, we further investigated changes in MR expression in both inner ear tissues and cultured vestibular epithelial cells derived from mice after rotatory stimulation or aldosterone administration, with or without ANP pretreatment. We found that MR expression was elevated in the vestibular tissues, particularly in the utricle of mice after rotatory stimulation (*P* < 0.01, Fig 2A-B), but not in the stria vascularis. Moreover, qRT-PCR and Western blot analysis revealed that both MR mRNA and protein levels in the inner ear tissues of mice were significantly elevated after rotatory stimulation and after aldosterone injection (*P* < 0.05 or 0.01, Fig 2C-G). Conversely, ANP pretreatment inhibited aldosterone injection- and rotatory stimulation-induced upregulation of MR expression at both mRNA and protein levels (*P* < 0.01, Fig 2C-G).

**Fig 3:**
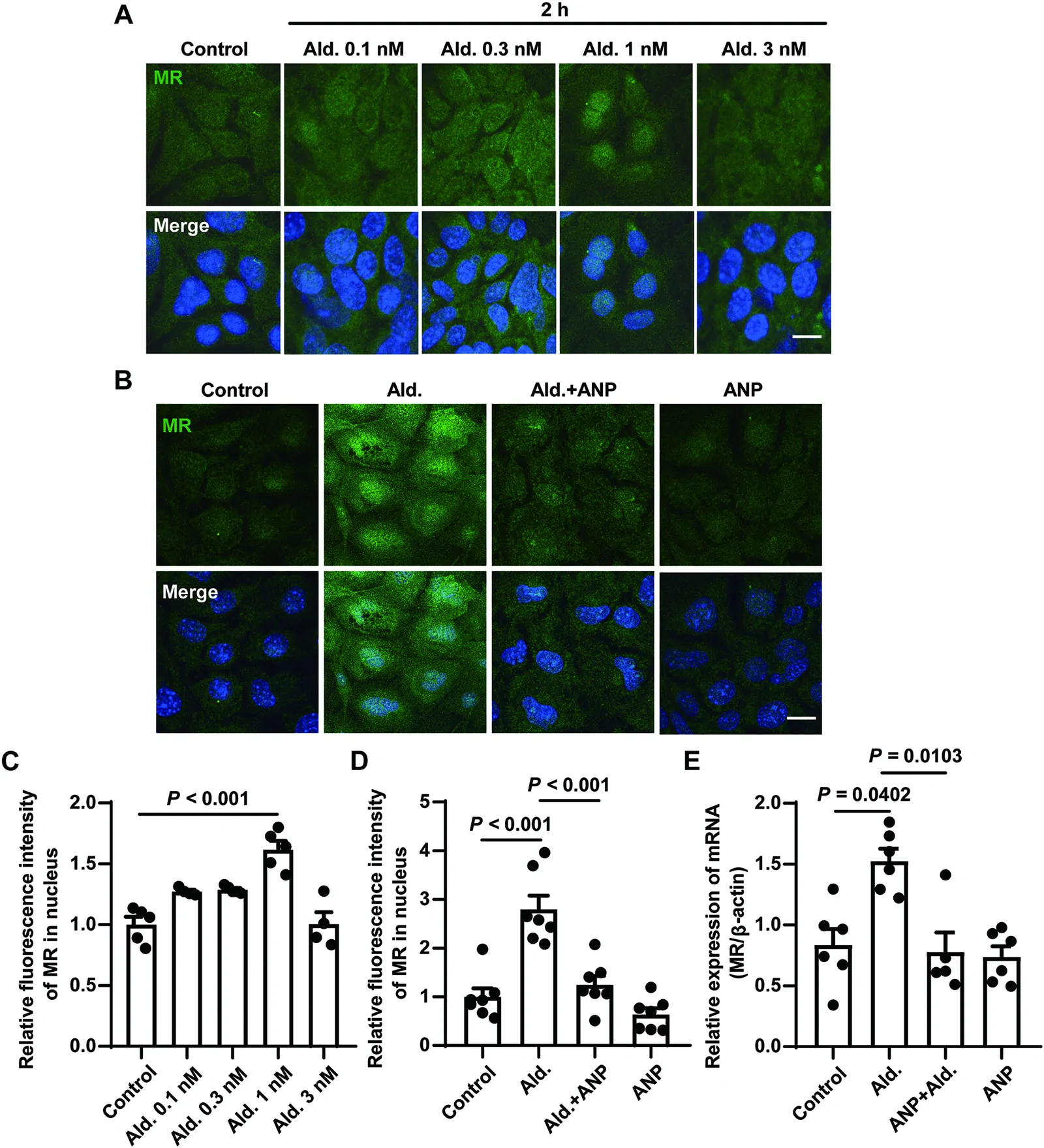
Aldosterone-induced upregulation of MR expression and nuclear translocation in cultured vestibular epithelial cells. A, Representative immunofluorescence images of MR expression in cultured vestibular epithelial cells treated with increasing concentrations of aldosterone. Scale bar, 10 µm. B, Representative immunofluorescence images of MR expression and nuclear translocation in cultured vestibular epithelial cells after aldosterone treatment, with or without ANP pretreatment. Scale bar, 10 µm. C, Quantification of the dose-dependent effect of aldosterone on the nuclear MR expression (n = 4-5 fields of view from 2 independent replicates). D, Quantification of nuclear MR immunofluorescence intensity (n = 7 fields of view from 2 independent replicates). E, MR mRNA levels in cultured vestibular epithelial cells after aldosterone treatment, with or without ANP pretreatment (n = 5-6 dishes from 2 independent replicates). Ald., aldosterone.

Furthermore, in an *in-vitro* study using cultured vestibular epithelial cells derived from mice, we found that aldosterone treatment dose-dependently upregulated MR expression, in particular in the nucleus (Fig 3A, 3C), suggesting an increased proportion of MRs translocating to nucleus. The effect of 1 nM aldosterone was most pronounced (*P* < 0.01, Fig 3A, 3C), and therefore this concentration was selected for subsequent experiments. Subsequently, we examined the effect of ANP on MR expression and nuclear translocation. ANP pretreatment significantly attenuated the 1 nM aldosterone-induced upregulation of MR expression and nuclear translocation (*P* < 0.01, Fig 3B, 3D). At the same time, MR mRNA levels in the vestibular epithelial cells after 1 nM aldosterone treatment and/or ANP pretreatment were also quantified. We found that aldosterone treatment upregulated MR mRNA level (*P* < 0.05, Fig 3E), whereas ANP pretreatment suppressed this aldosterone-induced upregulation (*P* < 0.05, Fig 3E).

### ANP suppresses the aldosterone-induced upregulation of the downstream target proteins ENaC and NKA in cultured vestibular epithelial cells

**Fig 4:**
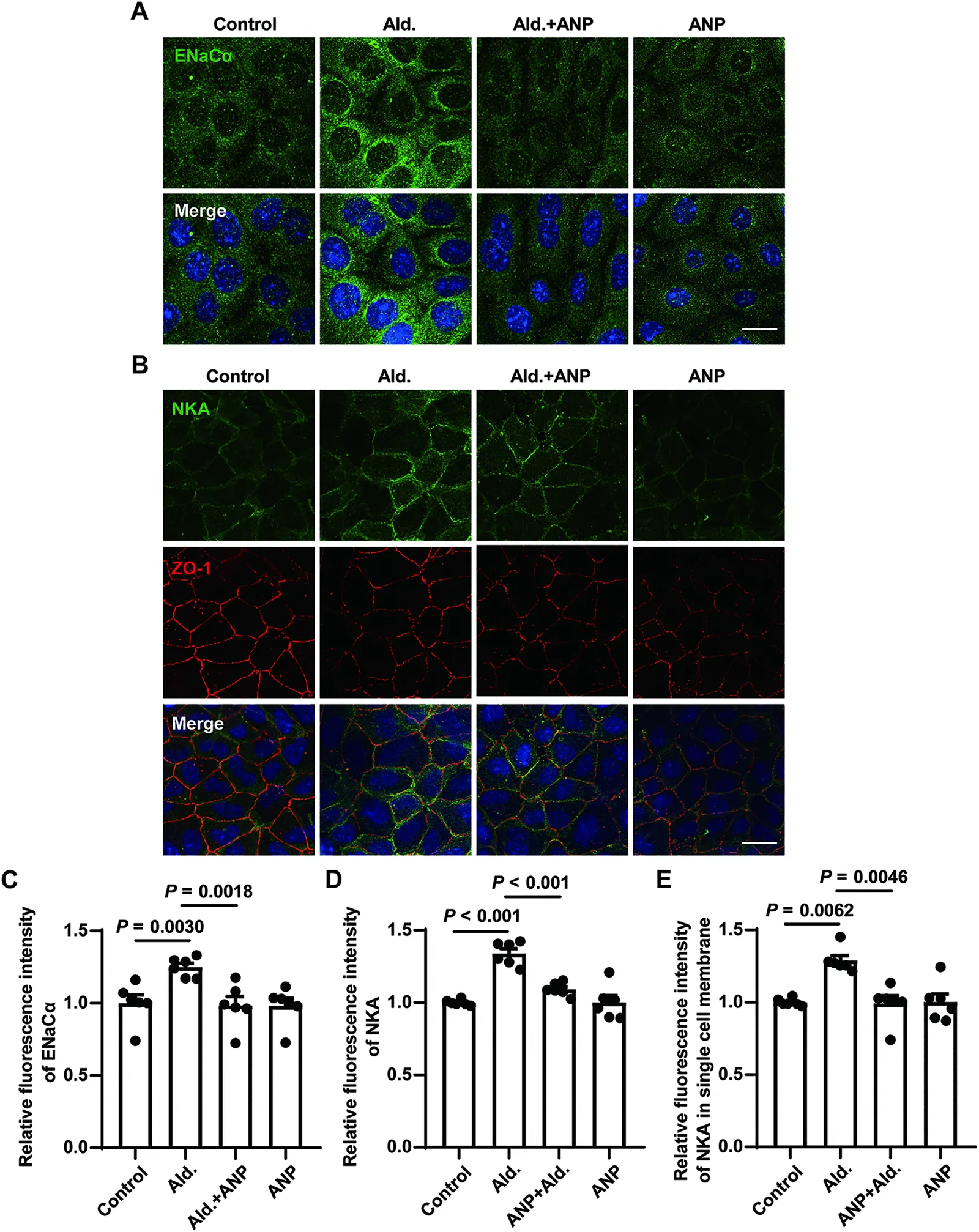
Aldosterone-induced upregulation of ENaC and NKA expression in cultured vestibular epithelial cells. A, Representative immunofluorescence images of ENaC expression in cultured vestibular epithelial cells after aldosterone treatment, with or without ANP pretreatment. Scale bar, 10 µm. B, Representative immunofluorescence images of NKA expression in cultured vestibular epithelial cells after aldosterone treatment, with or without ANP pretreatment. Scale bar, 10 µm. C, Quantification of ENaC immunofluorescence intensity (n = 6 fields of view from 2 independent replicates). D, Quantification of NKA immunofluorescence intensity (n = 6 fields of view from 2 independent replicates). E, Quantification of membranous immunofluorescence intensity of NKA co-localized with ZO-1 (n = 6 fields of view from 2 independent replicates). Ald., aldosterone.

The activities of both NKA and ENaC are well-established to be regulated by aldosterone as downstream target proteins [26,39]. We further investigated the effects of ANP on the expression of these two target proteins in cultured vestibular epithelial cells after aldosterone treatment. Consequently, aldosterone upregulated ENaC expression in the vestibular epithelial cells (*P* < 0.01, Fig 4A, 4C), whereas ANP pretreatment suppressed this aldosterone-induced upregulation of ENaC expression (*P* < 0.01, Fig 4A, 4C). In addition, aldosterone preferentially upregulated NKA expression at the membrane of the vestibular epithelial cells (*P* < 0.01, Fig 4B, 4D, and 4E), where it co-localized with the membrane protein ZO-1 (Fig 4B); however, ANP pretreatment suppressed this aldosterone-induced upregulation of membrane NKA expression (*P* < 0.01, Fig 4B, 4D, and 4E).

### ANP suppresses the aldosterone-induced upregulation of the intracellular fluorescence intensity of K^+^ and Na^+^ in cultured vestibular epithelial cells

**Fig 5:**
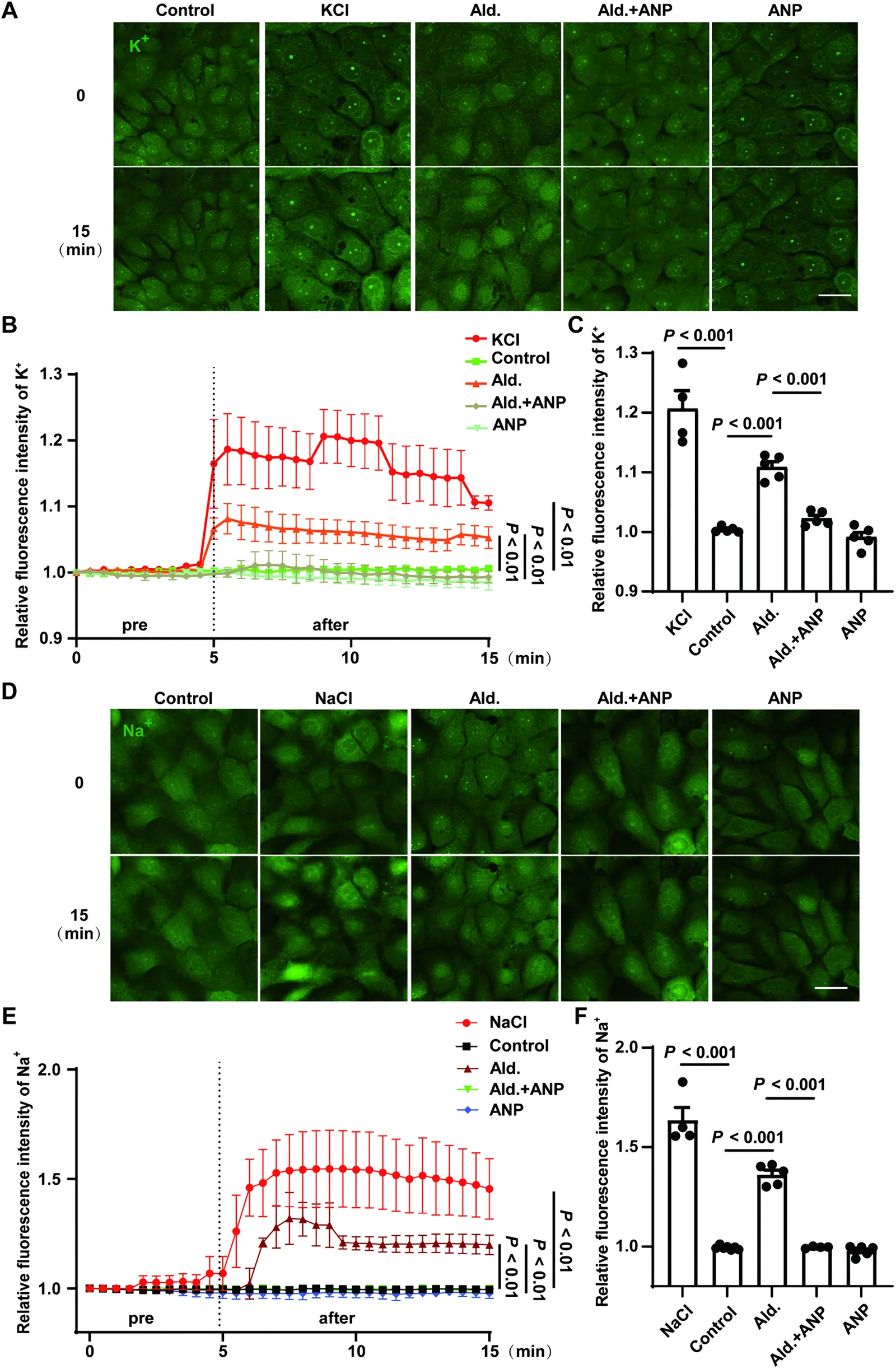
Aldosterone-induced elevation of intracellular K^+^ and Na^+^ fluorescence intensity in cultured vestibular epithelial cells. A, Representative images of intracellular K^+^ fluorescence intensity in cultured vestibular epithelial cells after different treatments. Scale bar, 25 µm. B, Quantification of time-dependent changes in K^+^ fluorescence intensity (n = 4-5 fields of view from 2 independent replicates). C, Quantification of intracellular K^+^ fluorescence intensity at 2 min after each treatment (n = 4-5 fields of view from 2 independent replicates). D, Representative images of intracellular Na^+^ fluorescence intensity in cultured vestibular epithelial cells after different treatments. Scale bar, 25 µm. E, Quantification of time-dependent changes in Na^+^ fluorescence intensity (n = 4-7 fields of view from 2 independent replicates). F, Quantification of intracellular Na^+^ fluorescence intensity at 2 min after each treatment (n = 4-7 fields of view from 2 independent replicates). Ald., aldosterone.

Furthermore, changes in intracellular K^+^ and Na^+^ of fluorescence intensity in cultured vestibular epithelial cells were examined after aldosterone treatment, with or without ANP pretreatment. Aldosterone applied to simulated endolymph fluid induced an increase in intracellular K^+^ fluorescence intensity in the vestibular epithelial cells (*P* < 0.01, Fig 5A-C); however, ANP pretreatment attenuated this aldosterone-induced increase in intracellular K^+^ fluorescence intensity (*P* < 0.01, Fig 5A-C). Similarly, aldosterone applied to an extracellular fluid increased intracellular Na^+^ fluorescence intensity in the vestibular epithelial cells (*P* < 0.01, Fig 5D-F); however, ANP pretreatment attenuated this aldosterone-induced increase in intracellular Na^+^ fluorescence intensity (*P* < 0.01, Fig 5D-F). All data are shown in S1 Table.

## Discussion

In the present study, we found that the motion sickness-provoking rotatory stimulation significantly induced an elevation in plasma aldosterone levels, whereas ANP pretreatment attenuated this elevation. The rotatory stimulation-induced elevation in plasma aldosterone levels observed in the present study is consistent with the findings of many previous studies [5–11]. Furthermore, intraperitoneal injection of aldosterone likewise induced signs similar to those of motion sickness in both guinea pigs and mice, including CTA behavior and an increase in the swim trajectory curvature, and ANP pretreatment attenuated these signs. Moreover, aldosterone injection induced an increase in the inner ear endolymph volume in guinea pigs similar to that induced by the motion sickness-provoking rotatory stimulation we previously reported [30]. The aldosterone-induced endolymph expansion in the inner ear in the present study is consistent with the findings of other studies [27–29]. ANP pretreatment in the present study attenuated the aldosterone-induced endolymph expansion in the inner ear of guinea pigs, an effect likewise consistent with the ANP-mediated attenuation observed following rotatory stimulation in our previous report [30]. These results suggest that rotatory stimulation-induced elevation in plasma aldosterone levels may cause the endolymph expansion in the inner ear, which may be involved in the induction of motion sickness; furthermore, ANP may attenuate the plasma aldosterone level elevation and the associated endolymph expansion, and consequently contribute to reducing the development of motion sickness. However, we still need further study to provide direct evidence to verify the causality of plasma aldosterone level elevation, endolymph expansion, and the development of motion sickness.

Owing to technical limitations, endolymph imaging of the vestibular structures could not be adequately performed in the present study; consequently, only changes in the endolymph volume of the cochlea were measured, and vestibular endolymph volume changes were not determined. However, as the endolymph compartments of the vestibule and cochlea are interconnected, an acute increase in the endolymph volume in the cochlea would affect the vestibule, at least through pressure transmission, and may thus lead to heightened vestibular sensitivity [40,41]. Therefore, the increase in endolymph volume induced by aldosterone injection in the present study or after rotatory stimulation in our previous study [30] may trigger vestibular autonomic responses, which bear resemblance to the clinical manifestations of Meniere’s disease, such as dizziness and prominent autonomic responses [42].

To elucidate the mechanisms underlying the aldosterone-induced endolymph expansion in the inner ear, its relationship to motion sickness, and the inhibitory action of ANP, we conducted further experiments and found that the MR expression was upregulated in the inner ear tissues after rotatory stimulation and after aldosterone injection, as well as in cultured vestibular epithelial cells after aldosterone treatment. However, ANP pretreatment suppressed MR expression in the animals subjected to rotatory stimulation or to aldosterone injection. In cultured vestibular epithelial cells, ANP pretreatment attenuated the aldosterone-induced upregulation of expression and nuclear accumulation of MRs. These findings may suggest that ANP not only suppresses aldosterone-induced upregulation of MR expression, but also inhibits the nuclear translocation of MRs. Moreover, we further investigated the effects of ANP on the downstream target proteins of the aldosterone-MR signaling pathway [24–26] and found that ANP pretreatment suppressed the aldosterone-induced upregulation of ENaC and NKA expression, as well as the membrane translocation of NKA in cultured vestibular epithelial cells. These results are consistent with previously reported effects of ANP on the inner ear [21] and the kidney [43]. Additional results from the present study revealed that aldosterone treatment induced an increase in intracellular K^+^ fluorescence intensity in the cultured vestibular epithelial cells incubated with a simulated endolymph fluid containing a high concentration of KCl. Aldosterone treatment likewise induced an increase in intracellular Na^+^ fluorescence intensity in the cultured vestibular epithelial cells incubated with an extracellular fluid containing a high concentration of NaCl. However, ANP pretreatment attenuated the aldosterone-induced increases in intracellular K^+^ and Na^+^ fluorescence intensity, suggesting that ANP-mediated suppression of aldosterone-induced ENaC and NKA expression and NKA membrane translocation may thereby maintain the ionic balance of the vestibular epithelial cells and consequently, ensure the water and electrolyte balance of the endolymph. Therefore, we speculate that ANP may exert its anti-motion sickness effects by attenuating rotatory stimulation-induced elevation in the plasma level of aldosterone and subsequently suppressing aldosterone-induced alterations in ionic homeostasis of the inner ear and the resultant increase in the endolymph volume.

ANP receptors have been identified in the inner ear [44,45], and preliminary investigations into the role of ANP in the inner ear have been conducted [46,47]. The present results provide direct evidence for a role of ANP in the modulation of inner ear endolymph homeostasis. Dzhoyashvili et al. reported that M-atrial natriuretic peptide significantly attenuated the furosemide-induced elevation in plasma aldosterone levels in spontaneously hypertensive rats [48]. A review by Kuwahara [49] indicates that ANP could directly suppress aldosterone synthesis in the zona glomerulosa of the adrenal cortex [50,51] and its secretion [52,53], and that ANP also acts as a corticotropin release-inhibiting factor to suppress corticotropin-releasing hormone (CRH) release within the hypothalamus and reduce adrenocorticotropic hormone release by inhibiting the pituitary response to CRH [54]. However, it remains unclear why ANP attenuated the elevation in plasma aldosterone levels after rotatory stimulation in the present study specifically, whether this reflects ANP directly inhibiting the synthesis and secretion of aldosterone in the adrenal cortex or whether ANP acts on hypothalamic or pituitary neurons that regulate adrenal gland function, or on more upstream central nuclei. In addition, as the ionic concentration of the endolymph currently cannot be monitored *in vivo* or measured *in vitro* owing to technical limitations, the exact changes in the concentration of Na^+^, K^+^ and other ions in the endolymph after rotatory stimulation, aldosterone administration, and ANP treatment remain undetermined. Therefore, numerous additional studies are warranted to elucidate the inner ear- and endolymph-related mechanisms underlying the anti-motion sickness action of ANP, as well as the potential CNS mechanisms involved.

Furthermore, the present study has several limitations. For example, the motion sickness model employed in the present study is a small animal model that differs substantially from the human condition, CTA and swimming behavior are surrogate measures of motion sickness-like responses rather than direct measures of motion sickness. Future studies using large animal models are recommended to evaluate the efficacy of candidate anti-motion sickness agents using more direct behavioral indicators, such as salivation and vomiting. Moreover, the current evidence for a causal relationship between the increased endolymph volume and the signs of motion sickness remains indirect; further experiments are warranted to determine whether directly inhibiting the increase in endolymph volume can suppress motion sickness, and whether endolymph expansion after rotatory stimulation is the primary determinant of motion sickness signs. ANP receptors and MRs are likewise expressed in the brain and many other tissues [55,56]. Accordingly, ANP formulations and MR antagonists may exert many effects on the CNS and other systems, potentially causing undesirable side effects when administered for the prevention of motion sickness, such as central side effects, cardiovascular and renal influences, and changes in endocrine system. Therefore, the development of ANP formulations and MR antagonists that do not cross the blood-brain barrier, and thus lack CNS effects, warrants consideration. However, except the central side effects, the side effects of these agents on other systems must be observed also in order to comprehensively evaluate their translational application prospects. In addition, a degree of inconsistency was observed between the results presented in Fig 2A-B and Fig 2C-D of the present study; additional immunohistochemical analyses using a larger number of animal samples are required to confirm these findings.

## Conclusions

The present results suggest that ANP may play an anti-motion sickness role by attenuating the rotatory stimulation-induced elevation in the plasma aldosterone level and by inhibiting the aldosterone effects on endolymph homeostasis via downregulation of downstream target protein expression and activity, thereby altering the ionic concentrations in the inner ear epithelial cells. However, further studies are needed to verify the causality of plasma aldosterone level elevation, changes in ion homeostasis and volume of endolymph in the inner ear, and the development of motion sickness. Therefore, the present study offers only preliminary evidence supporting the development of anti-motion sickness agents that target the ANP receptors (NPR-A) or aldosterone receptors (MRs) in the inner ear. Specifically, future anti-motion sickness drug development may consider NPR-A agonists, MR antagonists, or a combination thereof, all acting peripherally within the inner ear, as a strategy to circumvent the CNS side effects associated with currently available drugs. Nevertheless, side effects on other peripheral systems must be evaluated also in the process of translational application.

## Supporting information

S1 Fig This is a preliminary time-course experiment illustrating the promoting effect of aldosterone on endolymph volume in the inner ear of guinea pigs. A, Representative cochlear MRI images from guinea pigs at various time points after aldosterone injection at two different doses: a) Control; b) Ald. 1 h (100 μg/kg); c) Ald. 3 h (100 μg/kg); d) Ald. 1 h (31.32 μg/kg); e) Ald. 3 h (31.32 μg/kg); f) Ald. 5 h (31.32 μg/kg). Scale bar, 1 mm. B, Quantification of endolymph volume after aldosterone injection for individual animals.
(TIF)

S2 Fig This is an immunofluorescence evidence of cultured vestibular epithelial cells. A, Representative images of epithelial cell marker cytokeratin 7 (CK7) expression. The images below are enlarged versions of the areas enclosed by white boxes in the upper panels. Scale bar: 50 μm. B, Epithelial cells purity.
(TIF)

S3 Fig Original gel images of Western blot analysis. A, Original gel image showing the Western blot analysis of β-actin corresponding to Fig 2C. B, Original gel image showing the Western blot analysis of MR corresponding to Fig 2C. C, Original gel image showing the Western blot analysis of β-actin corresponding to Fig 2E. D, Original gel image showing the Western blot analysis of MR corresponding to Fig 2E.
(TIF)

S1 Table Original data supporting the Figs 1–5 are provided in the Excel file “Data-Fig 1 to Fig 5” with sheets labeled by figure number. (XLSX)

## References

[1] Keshavarz B, Golding JF. Motion sickness: Current concepts and management. Curr Opin Neurol. 2022;35(1):107–12. doi: 10.1097/WCO.0000000000001018 34839340

[2] Takov V, Tadi P. Motion Sickness. StatPearls [Internet]. Treasure Island (FL): StatPearls Publishing; 2026.

[3] Leung AKC, Hon KL. Motion sickness: An overview. Drugs Context. 2019;8:2019-9–4. doi: 10.7573/dic.2019-9-4 PMC7048153 32158479

[4] Koch A, Cascorbi I, Westhofen M, Dafotakis M, Klapa S, Kuhtz-Buschbeck JP. The neurophysiology and treatment of motion sickness. Dtsch Arztebl Int. 2018;115(41):687–96. doi: 10.3238/arztebl.2018.0687 30406755 PMC6241144

[5] Stalla GK, Doerr HG, Bidlingmaier F, Sippel WG, von Restorff W. Serum levels of eleven steroid hormones following motion sickness. Aviat Space Environ Med. 1985;56(10):995–9. 4062773

[6] Dai Y, Ji G, Huang Y, Sun X, Dai F. Changes of plasma endocrine hormone in pilots under Coriolis acceleration. Space Med Med Eng (Beijing). 1998;11(2):121–3. 11543228

[7] Grigoriev AI, Nichiporuk IA, Yasnetsov VV, Shashkov VS. Hormonal status and fluid electrolyte metabolism in motion sickness. Aviat Space Environ Med. 1988;59(4):301–5. 3370037

[8] Pei JS, Liu ZX, Yang TB, Tong BL, Yang YH, Jin WY. Changes of some hormones and inorganic salts in evoked motion sickness. Space Med Med Eng. 1995;8(1):7–11. doi: 10.16289/j.cnki.1002-0837.1995.01.003

[9] Drummer C, Norsk P, Heer M. Water and sodium balance in space. Am J Kidney Dis. 2001;38(3):684–90. doi: 10.1053/ajkd.2001.27765 11532707

[10] Norsk P. Renal adjustments to microgravity. Pflugers Arch. 2000;441(2-3 Suppl):R62–5. doi: 10.1007/s004240000332 11200982

[11] Demontis GC, Germani MM, Caiani EG, Barravecchia I, Passino C, Angeloni D. Human pathophysiological adaptations to the space environment. Front Physiol. 2017;8:547. doi: 10.3389/fphys.2017.00547 28824446 PMC5539130

[12] Furuta H, Mori N, Sato C, Hoshikawa H, Sakai S, Iwakura S, et al. Mineralocorticoid type I receptor in the rat cochlea: mRNA identification by polymerase chain reaction (PCR) and in situ hybridization. Hear Res. 1994;78(2):175–80. doi: 10.1016/0378-5955(94)90023-x 7982810

[13] Pitovski DZ, Drescher MJ, Drescher DG. High affinity aldosterone binding sites (type I receptors) in the mammalian inner ear. Hear Res. 1993;69(1–2):10–4. doi: 10.1016/0378-5955(93)90088-i 8226329

[14] Yao X, Rarey KE. Localization of the mineralocorticoid receptor in rat cochlear tissue. Acta Otolaryngol. 1996;116(3):493–6. doi: 10.3109/00016489609137879 8790754

[15] Sinha PK, Pitovski DZ. [3H]-aldosterone binding sites (type I receptors) in the lateral wall of the cochlea: distribution assessment by quantitative autoradiography. Acta Otolaryngol. 1995;115(5):643–7. doi: 10.3109/00016489509139380 8928636

[16] Ichimiya I, Adams JC, Kimura RS. Immunolocalization of Na, K -ATPase, Ca2 -ATPase, calcium-binding proteins, and carbonic anhydrase in the guinea pig inner ear. Acta Otolaryngol. 1994;114(2):167–76. doi: 10.3109/00016489409126037 8203199

[17] ten Cate WJ, Curtis LM, Rarey KE. Na,K-ATPase alpha and beta subunit isoform distribution in the rat cochlear and vestibular tissues. Hear Res. 1994;75(1–2):151–60. doi: 10.1016/0378-5955(94)90066-3 8071142

[18] McGuirt JP, Schulte BA. Distribution of immunoreactive alpha- and beta-subunit isoforms of Na,K-ATPase in the gerbil inner ear. J Histochem Cytochem. 1994;42(7):843–53. doi: 10.1177/42.7.8014467 8014467

[19] Zhong S-X, Hu G-H, Liu Z-H. Expression of ENaC, SGK1 and Nedd4 isoforms in the cochlea of guinea pig. Folia Histochem Cytobiol. 2014;52(2):144–8. doi: 10.5603/FHC.2014.0010 25007182

[20] Yamazaki M, Kim KX, Marcus DC. Sodium selectivity of Reissner’s membrane epithelial cells. BMC Physiol. 2011;11:4. doi: 10.1186/1472-6793-11-4 21284860 PMC3042420

[21] Luo Y, Xia Q, Xia Z, Tang Y. Atrial natriuretic peptide reduces the α-subunit of the epithelial sodium channel mRNA expression in the mouse stria vascularis. Biomed Rep. 2015;3(2):159–62. doi: 10.3892/br.2014.400 25798240 PMC4360879

[22] Kim SH, Park HY, Choi HS, Chung HP, Choi JY. Functional and molecular expression of epithelial sodium channels in cultured human endolymphatic sac epithelial cells. Otol Neurotol. 2009;30(4):529–34. doi: 10.1097/MAO.0b013e31819a8e0e 19300301

[23] Ferrary E, Bernard C, Teixeira M, Julien N, Bismuth P, Sterkers O, et al. Hormonal modulation of inner ear fluids. Acta Otolaryngol. 1996;116(2):244–7. doi: 10.3109/00016489609137833 8725524

[24] Al-Mana D, Ceranic B, Djahanbakhch O, Luxon LM. Hormones and the auditory system: A review of physiology and pathophysiology. Neuroscience. 2008;153(4):881–900. doi: 10.1016/j.neuroscience.2008.02.077 18440718

[25] Kamynina E, Staub O. Concerted action of ENaC, Nedd4-2, and Sgk1 in transepithelial Na(+) transport. Am J Physiol Renal Physiol. 2002;283(3):F377–87. doi: 10.1152/ajprenal.00143.2002 12167587

[26] Feraille E, Dizin E. Coordinated control of ENaC and Na+,K+-ATPase in renal collecting duct. J Am Soc Nephrol. 2016;27(9):2554–63. doi: 10.1681/ASN.2016020124 27188842 PMC5004664

[27] Dunnebier EA, Segenhout JM, Wit HP, Albers FW. Two-phase endolymphatic hydrops: A new dynamic guinea pig model. Acta Otolaryngol. 1997;117(1):13–9. doi: 10.3109/00016489709117984 9039474

[28] Qin L, Zhang B, Wang Q, Li D, Luo X, Zhong S. Effect of aldosterone on cochlear Af9 expression and hearing in guinea pig. Acta Otolaryngol. 2017;137(9):903–9. doi: 10.1080/00016489.2017.1309681 28399691

[29] Zhong S, Zhang B, Qin L, Wang Q, Luo X. Aldosterone inhibits Dot1l expression in guinea pig cochlea. Eur J Med Res. 2023;28(1):26. doi: 10.1186/s40001-023-00994-y 36639782 PMC9838020

[30] Xu L-H, Ge J-G, Xiao S-F, Lu Q-C, Ji W, Ma Y-Q, et al. Atrial natriuretic peptide alleviates motion sickness potentially through regulating endolymph volume in the inner ear increased by arginine vasopressin. Neuroendocrinology. 2024;114(8):786–98. doi: 10.1159/000539586 38815558

[31] Crampton GH, Lucot JB. A stimulator for laboratory studies of motion sickness in cats. Aviat Space Environ Med. 1985;56(5):462–5. 4004682

[32] Li X, Jiang Z-L, Wang G-H, Fan J-W. Plasma vasopressin, an etiologic factor of motion sickness in rat and human? Neuroendocrinology. 2005;81(6):351–9. doi: 10.1159/000088991 16230861

[33] Huang Q, Liao XW, Zhai SJ, Liu YC, Yin SH. Aldosterone-induced αENaC expression is associated with NF-κB activity in guinea pig cochlea. Chin J Otol. 2020;18(1):133–8. doi: 10.3969/j.issn.1672-2922.2020.01.023

[34] Frisina RD, Ding B, Zhu X, Walton JP. Age-related hearing loss: Prevention of threshold declines, cell loss and apoptosis in spiral ganglion neurons. Aging (Albany NY). 2016;8(9):2081–99. doi: 10.18632/aging.101045 27667674 PMC5076453

[35] Wu Y, Zhang Y, Xie B, Abdelgawad A, Chen X, Han M, et al. RhANP attenuates endotoxin-derived cognitive dysfunction through subdiaphragmatic vagus nerve-mediated gut microbiota-brain axis. J Neuroinflammation. 2021;18(1):300. doi: 10.1186/s12974-021-02356-z 34949194 PMC8697447

[36] Ohbayashi H, Suito H, Takagi K. Compared effects of natriuretic peptides on ovalbumin-induced asthmatic model. Eur J Pharmacol. 1998;346(1):55–64. doi: 10.1016/s0014-2999(98)00014-4 9617752

[37] Wang F, Lyu H, Zhao M, Sha Y, Zhang F, Cheng Y, et al. Assessment of cochlea endolymphatic hydrops using 3-D FLAIR and 3-D real IR sequence in guinea pigs via 3T mri after intratympanic gadolinium: A histopathological comparison. Otol Neurotol. 2017;38(4):585–90. doi: 10.1097/MAO.0000000000001331 28072657

[38] Marano RJ, Redmond SL. In vitro cultured primary cells from a human utricle explant possesses hair cell like characteristics. J Mol Histol. 2011;42(4):365–70. doi: 10.1007/s10735-011-9333-7 21660457

[39] Pearce D, Manis AD, Nesterov V, Korbmacher C. Regulation of distal tubule sodium transport: Mechanisms and roles in homeostasis and pathophysiology. Pflugers Arch. 2022;474(8):869–84. doi: 10.1007/s00424-022-02732-5 35895103 PMC9338908

[40] Rey-Martinez J, Altuna X, Cheng K, Burgess AM, Curthoys IS. Computing endolymph hydrodynamics during head impulse test on normal and hydropic vestibular labyrinth models. Front Neurol. 2020;11:289. doi: 10.3389/fneur.2020.00289 32390929 PMC7193182

[41] Soriano-Reixach MM, Rey-Martinez J, Altuna X, Curthoys I. Enhanced eye velocity with backup saccades in vHIT tests of a Menière disease patient: A case report. Front Surg. 2021;8:727672. doi: 10.3389/fsurg.2021.727672 34957197 PMC8692282

[42] Wu Y, Ling X, Song N, Yan S, Wang W, Yang X, et al. Comparison of clinical characteristics and vestibular function test results in patients with vestibular migraine and Menière’s disease. Braz J Otorhinolaryngol. 2023;89(4):101274. doi: 10.1016/j.bjorl.2023.05.001 37331235 PMC10300291

[43] Theilig F, Wu Q. ANP-induced signaling cascade and its implications in renal pathophysiology. Am J Physiol Renal Physiol. 2015;308(10):F1047-55. doi: 10.1152/ajprenal.00164.2014 25651559 PMC4436998

[44] Lamprecht J, Meyer zum Gottesberge AM. The presence and localization of receptors for atrial natriuretic peptide in the inner ear of the guinea pig. Arch Otorhinolaryngol. 1988;245(5):300–1. doi: 10.1007/BF00464636 2854462

[45] Long L, Tang Y, Xia Q, Xia Z, Liu J. Detection of atrial natriuretic peptide receptor in the labyrinth of the mouse inner ear. Neuro Endocrinol Lett. 2008;29(4):577–80. 18766159

[46] Yoon YJ, Lee EJ, Hellstrom S, Kim JS. Atrial natriuretic peptide modulates auditory brainstem response of rat. Acta Otolaryngol. 2015;135(12):1293–7. doi: 10.3109/00016489.2015.1073354 26245816

[47] Rachel JD, Dziadziola JK, Quirk WS. Atrial natriuretic peptide participates in the regulation of vestibular blood flow. Hear Res. 1995;89(1–2):181–6. doi: 10.1016/0378-5955(95)00135-3 8600124

[48] Dzhoyashvili NA, Iyer SR, Chen HH, Burnett JCJ. MANP (M-Atrial Natriuretic Peptide) reduces blood pressure and furosemide-induced increase in aldosterone in hypertension. Hypertension. 2022;79(4):750–60. doi: 10.1161/HYPERTENSIONAHA.121.18837 PMC8916975 35045724

[49] Kuwahara K. The natriuretic peptide system in heart failure: Diagnostic and therapeutic implications. Pharmacol Ther. 2021;227:107863. doi: 10.1016/j.pharmthera.2021.107863 33894277

[50] Chartier L, Schiffrin E, Thibault G, Garcia R. Atrial natriuretic factor inhibits the stimulation of aldosterone secretion by angiotensin II, ACTH and potassium in vitro and angiotensin II-induced steroidogenesis in vivo. Endocrinology. 1984;115(5):2026–8. doi: 10.1210/endo-115-5-2026 6092045

[51] De Léan A, Racz K, Gutkowska J, Nguyen TT, Cantin M, Genest J. Specific receptor-mediated inhibition by synthetic atrial natriuretic factor of hormone-stimulated steroidogenesis in cultured bovine adrenal cells. Endocrinology. 1984;115(4):1636–8. doi: 10.1210/endo-115-4-1636 6090110

[52] Nishikimi T, Maeda N, Matsuoka H. The role of natriuretic peptides in cardioprotection. Cardiovasc Res. 2006;69(2):318–28. doi: 10.1016/j.cardiores.2005.10.001 16289003

[53] Rao S, Pena C, Shurmur S, Nugent K. Atrial natriuretic peptide: structure, function, and physiological effects: A narrative review. Curr Cardiol Rev. 2021;17(6):e051121191003. doi: 10.2174/1573403X17666210202102210 33530911 PMC8950497

[54] McCann SM, Antunes-Rodrigues J, Franci CR, Anselmo-Franci JA, Karanth S, Rettori V. Role of the hypothalamic pituitary adrenal axis in the control of the response to stress and infection. Braz J Med Biol Res. 2000;33(10):1121–31. doi: 10.1590/s0100-879x2000001000001 11004712

[55] Pandey KN. Guanylyl cyclase/natriuretic peptide receptor-A: Identification, molecular characterization, and physiological genomics. Front Mol Neurosci. 2023;15:1076799. doi: 10.3389/fnmol.2022.1076799 36683859 PMC9846370

[56] Martinerie L, Hani I, Perrot J, Viengchareun S. Tissue-specific expression and regulation of the mineralocorticoid receptor during development. J Endocrinol. 2026;268(1):e250265. doi: 10.1530/JOE-25-0265 41543024

