# Supplementary material for: Atrial natriuretic peptide may inhibit motion sickness through reducing aldosterone-induced increase in endolymph volume of the inner ear
Source: PLoS One. 2026 Sep 25;21(9):e0359426. doi: 10.1371/journal.pone.0359426 (PMC13614638; doi:10.1371/journal.pone.0359426)
Supplement: Supplementary file 1 [file pone.0359426.s006.docx]

**Response to Reviewers**

**Reviewer #1:**

1. What are some examples of central side effects of present drugs?

**Response:** Thank you for your suggestion, we have listed some examples of central side effects of present drugs in the section of “Introduction” in line 47-48 of page 3.

2. What are some limitations of the study?

**Response:** We have introduced limitations of the present study in the section of “Discussion” (line 383-386, page 14; line 440-455, page 16).

3. Page 4 died (dying)

**Response:** Thank you for your suggestion, we have revised (line 107, page 5).

4. What are some possible side effects of Atrial Natriuretic Peptides levels?

**Response:** According to literature (Ref. 55, Front Mol Neurosci. 2023;4:15:1076799), the receptor (NPRA) for atrial natriuretic peptide (ANP) is expressed in the heart, kidney, adrenal gland, brain, lung, ovary, testis, thymus, vasculature, and pituitary gland, and ANP may thus play diverse roles, which may constitute side effects of ANP especially those side effects on the central nervous system when we use it for the prevention of motion sickness. This issue has been discussed in the section of “Discussion” (line 449-451, page 16).

5. Did you explore other category of drugs such as aldosterone inhibitors such as "Eplerenome" which has fewer side effects that would target the ANP receptors (NPR-A) or aldosterone receptors (MRs) in the inner ear?

**Response:** Yes, we have investigated the anti-motion sickness effects of three generations of mineralocorticoid receptor antagonists including spironolactone (i.p.), eplerenome (intratympanic microinjection, i.t.), and esaxerenone (i.t.), and found that these three inhibitors of MRs all reduced the conditioned taste aversion (CTA) behaviours in rats induced by rotatory stimulation and by aldosterone injection. In addition, intragastric administration of eplerenome inhibited rotatory stimulus-induced and aldosterone injection-induced CTA behaviours, abnormalities in swimming behavior, and endolymph volume expansion in guinea pigs. These results will be reported recently in new paper in detail. The anti-motion sickness of ANP has been reported in our previous report (Ref. 30).

6. Where there any combination treatments considered?

**Response:** Yes, many people have already considered the combinative use of drugs, such as the current combination of anticholinergic drugs and sympathomimetic drugs, or antihistamines and sympathomimetic drugs that have been used to prevent motion sickness (Refs. 1-4 in the manuscript), which has been discussed in the section of “Discussion” of the revised manuscript, i.e., we could consider a combination use of ANP formulations and MR antagonist in the future (line 463-466, page 17).

**Reviewer #2:**

This manuscript investigates the hypothesis that atrial natriuretic peptide (ANP) inhibits motion sickness by reducing aldosterone-induced endolymph volume expansion in the inner ear. The authors use a combination of in vivo (guinea pig and mouse) and in vitro (primary vestibular epithelial cell culture) approaches to demonstrate that: (1) rotatory stimulation increases plasma aldosterone levels; (2) aldosterone injection mimics motion sickness symptoms and increases endolymph volume; (3) ANP pretreatment blocks these effects; (4) ANP reduces aldosterone-induced expression and nuclear translocation of mineralocorticoid receptors (MR); (5) ANP inhibits aldosterone-induced expression of ENaC and Na⁺-K⁺-ATPase; and (6) ANP attenuates aldosterone-induced increases in intracellular K⁺ and Na⁺ concentrations in vestibular epithelial cells. The topic is interesting and potentially relevant to understanding the peripheral mechanisms of motion sickness.

1. The behavioral index of motion sickness (conditioned taste aversion, CTA) is indirect. While CTA is a validated measure of nausea/malaise in rodents, the authors do not provide any validation that aldosterone-induced CTA is truly equivalent to motion sickness induced by rotatory stimulation. Please provide a direct comparison showing that rotatory stimulation produces CTA under the same conditions, or use additional behavioral measures (e.g., pica, conditioned gaping) to support the interpretation.

**Response:** Yes, we have investigated the effect of ANP on rotatory stimulus-induced behavioural changes in CTA and vestibular function, and increase in the inner ear endolymph volume (published recently, Ref. 30 of this manuscript), and introduced these results in the revised manuscript “Results” section (line 312-314, page 12), and this anti-motion sickness effect of ANP is also mentioned in the “Introduction” section (line 83-84, page 4).

2. The doses of ANP (400 μg/kg for guinea pigs, 500 μg/kg for mice) and aldosterone (100 μg/kg for guinea pigs, 114 μg/kg for mice) are not justified. No dose-response experiments are shown. Please provide dose-response curves for both drugs or cite published literature that established these doses.

**Response:** The doses of aldosterone were based on literature reports (Refs. 33-34) and our recent experiments that has not been reported yet (S1 Fig), this issue has been introduced in the revised manuscript “Materials and Methods” section (line 133-134, page 6). The doses of ANP were based on our previous study (Ref. 30) and literature reports (Refs. 35-36), this problem has been introduced in the revised manuscript “Materials and Methods” section (line 140-141, page 6).

3. The swimming test curvature measurement (Figure 1B-C) is presented as a measure of vestibular function, but the methods do not specify how many trials per animal were analyzed or whether the experimenter was blinded to treatment groups. Please add this information.

**Response:** Thank you for reminding us, we have added this information in the revised manuscript “Materials and Methods” section (line 154-155, page 6).

4. The sample sizes (n numbers) are not reported for most experiments. Please provide exact n values for each experiment in the figure legends (e.g., number of animals per group for in vivo studies, number of culture replicates for in vitro studies).

**Response:** Thank you for your suggestion, we have added this information in the revised manuscript “Figure legends” section (page 24-page 25).

5. In Figure 1A, the plasma aldosterone level after rotatory stimulus is shown with n=? and the ANP pretreatment group shows a reduction compared to rotatory alone. However, the rotatory + ANP group is not compared to a non-rotated ANP control. Please include a control group receiving ANP without rotatory stimulus to rule out direct effects of ANP on baseline aldosterone.

**Response:** In fact, in Figure 1A, the group “ANP” is also a control group receiving ANP without rotatory stimulus, and there is no statistical difference between the groups “Control” and “ANP”. There is also no statistical difference between the groups “Rotation+ANP” and “ANP”. Therefore, they were not labeled.

6. The MRI measurement of endolymph volume (Figure 1F-G) is a key endpoint, but the methods are insufficiently described. How was the boundary between endolymph and perilymph determined? Was the analysis performed blinded? What is the intra- and inter-rater reliability? Please add this information. Also, the representative images in Figure 1F appear to have different contrast/quality between groups; please provide images from all animals or state that these are representative.

**Response:** Thank you for your suggestion. In Figure 1F, we have added dashed circles to outline the endolymph range, revised the sections of “Measurement of endolymph volume” (line 179-189, page 7) and “Figure legends” (line 656-658, page 24), and stated that these are representative cochlear MRI images from each experimental group of guinea pigs.

7. The immunofluorescence images in Figure 2A show MR expression in the utricle, saccule, and stria vascularis, but the quantification (Figure 2B) shows a statistically significant increase only in the utricle after rotatory stimulus. The authors then performed Western blot on whole inner ear tissue (Figure 2C-F). Since the increase appears to be region-specific, whole-tissue Western blot may dilute the effect. Please discuss this limitation or provide Western blot data from dissected utricle tissue.

**Response:** Yes, you are right. According to your suggestion, we have revised the manuscript and stated this limitation in the “Discussion” section (line 453-455, page 16).

8. The primary culture of vestibular epithelial cells is described, but the methods do not include characterization of these cells (e.g., expression of epithelial markers, hair cell vs. supporting cell markers). Please provide immunofluorescence evidence of epithelial identity (e.g., cytokeratin, ZO-1) and purity.

**Response:** According to your suggestion, we have added a new figure (S2 Fig) in the supplementary figures to show immunofluorescence evidence of epithelial cells (cytokeratin 7, CK7) and the purity.

9. In Figure 3A-B, the authors show a dose-dependent increase in MR nuclear localization with aldosterone (0.1-100 nM). However, the data are presented only as images; quantification is missing for the dose-response (only shown for 1 nM in later panels). Please add a bar graph showing the quantification of nuclear MR fluorescence across all doses tested.

**Response:** In fact, Figure 3A shows image examples of a dose-dependent increase in MR nuclear localization with aldosterone (0.1-3 nM), and Figure 3B is a bar graph showing the quantification of nuclear MR fluorescence dose-response (0.1-3 nM aldosterone) with statistical analysis.

10. The claim that ANP "inhibits the binding of MRs to aldosterone and their entry into the nucleus" (Discussion, lines 320-321) is not directly supported. The data show reduced MR expression and nuclear localization, but not reduced binding. Please rephrase to state that ANP reduces MR protein level and nuclear accumulation, without claiming direct competition for binding.

**Response:** Yes, you are right. We have revised this description of the manuscript “Discussion” section (line 402, page 15) according to your suggestion.

11. The measurement of intracellular K⁺ and Na⁺ concentrations (Figure 5) uses fluorescent dyes, but the methods do not describe calibration of fluorescence intensity to absolute ion concentration. The data are presented as "relative fluorescence intensity," not actual concentrations. Please revise the text and figures to clearly state that these are relative fluorescence changes, not absolute concentrations. Alternatively, provide calibration curves.

**Response:** Yes, we have revised the text of result description and figure 5 legend to clearly state that these are relative fluorescence intensity changes (line 352-362, page 13; line 408-413, page 15; line 699-708, page 25).

12. The experimental timeline for aldosterone injection and MRI (3h after aldosterone) is based on an unpublished or unspecified rationale. Please provide a time-course experiment showing the peak effect of aldosterone on endolymph volume, or cite literature supporting the 3h time point.

**Response:** Thank you for reminding us, we have added a new figure (S1 Fig) in the supplementary figures to present a time-course experiment showing the peak effect on endolymph volume of aldosterone at two doses.

13. The authors state that "ANP could directly suppress aldosterone production in the adrenal cortex" (Discussion, lines 357-359) but provide no data to support this. This is speculation. Please remove or provide evidence (e.g., adrenal gland measurements or in vitro adrenal cell studies).

**Response:** Yes, this is the conclusion of the research reports in the literature (Refs. 49-54, line 425-429, page 15), not from the present study, we only discussed with a description (line 429-433, page 15-16). Therefore, we did not revise this content of the “Discussion” section.

14. The title states "may inhibit motion sickness," which is appropriately cautious, but the abstract and conclusion state stronger claims (e.g., "ANP could play an anti-motion sickness role"). Please harmonize the language throughout to be consistent with the level of evidence (correlative, not causative).

**Response:** Yes, you are right. According to your suggestion, we have revised the relevant expressions in the manuscript (line 35, page 2; line 381, page 14; line 417, page 15; line 457, page 16).

15. The authors did not test whether ANP alone (without aldosterone or rotatory stimulus) affects endolymph volume or behavior. Please add these control groups or state their absence as a limitation.

**Response:** Similar to above question 5 of yours, in fact, in Figure 1F-G, the group “ANP” is also a control group receiving ANP without aldosterone injection, and there is no statistical difference between the groups “Control” and “ANP”. There is also no statistical difference between the groups “Ald.+ANP” and “ANP”.

16. The manuscript lacks a direct test of whether blocking MR (e.g., with spironolactone or eplerenone) mimics the effects of ANP on motion sickness. Such an experiment would strengthen the mechanistic conclusion. Please add this or discuss as a future direction.

**Response:** Similar to question 5 of above Reviewer #1, we have investigated the anti-motion sickness effects of three generations of mineralocorticoid receptor antagonists including spironolactone (i.p.), eplerenome (intratympanic microinjection, i.t.), and esaxerenone (i.t.), and found that these three inhibitors of MRs all reduced the conditioned taste aversion (CTA) behaviours in rats induced by rotatory stimulus and by aldosterone injection. In addition, intragastric administration of eplerenome inhibited rotatory stimulus-induced and aldosterone injection-induced CTA behaviours, abnormalities in swimming behavior, and endolymph volume expansion in guinea pigs. These results will be reported recently in new paper in detail. We have added related content as a future direction in the section “Conclusions” of the revised manuscript (line 463-466, page 17).

17. More recent advance for peptides must be introduced by incorporating all suggested references into the revised manuscript. In particular, peptide or peptide based therapeutics, which have been recognized as treasure trove for drug discovery and some of them have shown significant potency for regenerative medicine (PMID: 41017403, 40910702, 39479995, 36855153, https://www.zoores.ac.cn/article/doi/10.24272/j.issn.2095-8137.2025.499, https://www.zoores.ac.cn/article/doi/10.24272/j.issn.2095-8137.2026.076, https://doi.org/10.1016/j.cej.2026.173231).

**Response:** Thank you so much for your kind suggestion. However, papers you suggested are not relevant to our work, very sorry, we cannot cite them.

**Reviewer #3:**

General assessment

This manuscript investigates the potential role of aldosterone in motion sickness and proposes that atrial natriuretic peptide (ANP) suppresses motion sickness by reducing aldosterone-induced endolymph expansion in the inner ear. The study combines behavioral analyses, MRI assessment of endolymph volume, molecular analyses, and in vitro vestibular epithelial cell experiments. The topic is interesting and clinically relevant because it links endocrine regulation, inner ear fluid homeostasis, and vestibular function. The experiments appear technically sound, and the findings are generally consistent across different experimental approaches.

Overall, I consider this to be a well-designed study. However, several aspects of the interpretation should be revised to avoid overstatement. The data support an association between aldosterone signaling, endolymphatic regulation, and motion sickness-like behaviors, but they do not fully establish that endolymph expansion is the primary causal mechanism underlying motion sickness. These issues can be addressed by revision of the text rather than by additional experiments. Therefore, I recommend "Minor Revision".

-Major comments

1. Interpretation of motion sickness

The central conclusion of the manuscript should be interpreted more cautiously. Although the study demonstrates that aldosterone administration induces conditioned taste aversion (CTA), abnormalities in swimming behavior, and endolymph expansion, these findings do not conclusively demonstrate that endolymph expansion is the primary mechanism underlying motion sickness.

CTA is widely accepted as a surrogate behavioral index in rodent motion sickness research; however, it remains an indirect measure of motion sickness-like behavior rather than motion sickness itself. Likewise, abnormalities in swimming trajectory indicate vestibular dysfunction but are not specific for motion sickness. Therefore, I recommend that the authors clearly distinguish between association and causation and moderate statements suggesting that endolymph expansion directly causes motion sickness.

**Response:** Yes, your suggestions are constructive. We have discussed these problems in the revised manuscript (line 440-448, page 16).

2. Discussion of endolymphatic hydrops and current clinical evidence

The Discussion would benefit from placing the present findings into the context of recent clinical studies. Gadolinium-enhanced inner ear MRI has demonstrated that the degree of endolymphatic hydrops does not always correlate with symptom severity or disease activity in Ménière's disease. Therefore, although the present study suggests that aldosterone signaling influences endolymph volume, it should also be discussed that endolymph expansion may represent one component of vestibular pathophysiology rather than the sole determinant of vestibular symptoms.

**Response:** Yes, you are right, endolymphatic hydrop is one of the main pathophysiological changes of Ménière's disease, not the sole determinant of vestibular symptoms. In the same way, at present stage we cannot conclude that endolymph expansion after rotatory stimulus or after aldosterone use is the sole determinant of motion sickness symptoms. According to your suggestion, we have discussed in the section “Discussion” of the revised manuscript (line 444-448, page 16), we have to perform more experiments to verify this issue.

3. MRI evaluation of endolymph volume

The MRI measurements were performed in the cochlear compartment, whereas the functional outcomes mainly reflect vestibular function. Please discuss this limitation and explain the rationale for extrapolating cochlear endolymph changes to vestibular pathophysiology.

In addition, please clarify whether MRI measurements were performed in a blinded manner, how the regions of interest were selected, and whether measurement reproducibility (inter- or intra-observer variability) was evaluated.

**Response:** Thank you for your suggestions. According to your first suggestion, we have revised the “Discussion” section and pointed out this limitation (line 383-392, page 14). In addition, according to your second suggestion, we have revised the section “Measurement of endolymph volume” (line 179-189, page 7).

4. Characterization of cultured vestibular epithelial cells

Please clarify the cellular composition of the cultured vestibular epithelial cells. Information regarding cell identification or characterization would improve interpretation of the molecular findings involving MR, ENaC, and Na-K-ATPase.

**Response:** Yes, this is the same issue as the question 8 of above Reviewer #2. According to your suggestion, we have added a new figure (S2 Fig) in the supplementary figures to show immunofluorescence evidence of epithelial cells (cytokeratin 7, CK7) and the purity.

-Minor comments

1. The manuscript would benefit from careful English language editing to improve grammar and readability.

**Response:** Yes, we have performed careful English language editing of the manuscript and marked changes with red color.

2. Please provide the exact sample size (n) in each figure legend.

**Response:** According to your suggestion, we have added this information in the revised manuscript “Figure legends” section (page 24-25).

3. Although the Data Availability Statement states that all data are included in the manuscript, the underlying raw data supporting the figures (individual measurements underlying the summary statistics) are not available. To comply with the PLOS Data Policy, these data should be provided as supplementary files or deposited in an appropriate public repository.

**Response:** According to your suggestion, original raw data supporting the Figs 1-5 have been listed in the S1 Table in the section “Supporting information” of the revised manuscript.
