# Supplementary material for: Atrial natriuretic peptide may inhibit motion sickness through reducing aldosterone-induced increase in endolymph volume of the inner ear
Source: PLoS One. 2026 Sep 25;21(9):e0359426. doi: 10.1371/journal.pone.0359426 (PMC13614638; doi:10.1371/journal.pone.0359426)
Supplement: Supplementary file 2 [file pone.0359426.s007.docx]

**Response to Reviewers**

Minor revisions:

1. Further moderate mechanistic statements throughout the Discussion and Conclusions to avoid implying causality where only associative evidence has been demonstrated.

**Response:** Yes, according to this suggestion, we have revised related descriptions in the sections of “Discussion” (line 380, line 382-384, and line 392, page 14; line 418-419, page 15) and “Conclusions” (line 470-472, page 17).

2. Continue to emphasize that conditioned taste aversion (CTA) and swimming behavior are surrogate measures of motion sickness-like responses rather than direct measures of motion sickness.

**Response:** Yes, according to this suggestion, we have added new descriptions in the sections of “Materials and methods” (line 129-130, page 5; line 155-156, page 6) and “Discussion” (line 445-446, page 16).

3. Briefly expand the discussion on the translational limitations of systemic ANP administration, including its potential cardiovascular and endocrine effects.

**Response:** Yes, according to this suggestion, we have revised related descriptions in the sections of “Discussion” (line 453-457, line 459-461, page 16) and “Conclusions” (line 477-478, page 17).

4. Perform one final careful English language and typographical review to improve readability and consistency.

**Response:** We have completed the final careful English language editing including correction of typing errors, deletion of some abbreviations that were not repeatedly used, such as ACTH, BCA, ECL, FBS, HBSS, PVDF, RAAS, and ROI, and modification of some descriptions throughout the entire text.

 Moreover, to help us prepare publication quality figures, and to ensure our figures meet the technical requirements, we have reviewed the figure guidelines, and checked and adjusted the mismatched figures online using PLOS’s free figure tool, NAAS.
